# How does CHD4 slide nucleosomes?

**DOI:** 10.1042/BST20230070

**Published:** 2024-09-02

**Authors:** Xavier J. Reid, Yichen Zhong, Joel P. Mackay

**Affiliations:** School of Life and Environmental Sciences, University of Sydney, Darlington, NSW 2006, Australia

**Keywords:** CHD4, chromatin, enzyme activity

## Abstract

Chromatin remodelling enzymes reposition nucleosomes throughout the genome to regulate the rate of transcription and other processes. These enzymes have been studied intensively since the 1990s, and yet the mechanism by which they operate has only very recently come into focus, following advances in cryoelectron microscopy and single-molecule biophysics. CHD4 is an essential and ubiquitous chromatin remodelling enzyme that until recently has received less attention than remodellers such as Snf2 and CHD1. Here we review what recent work in the field has taught us about how CHD4 reshapes the genome. Cryoelectron microscopy and single-molecule studies demonstrate that CHD4 shares a central remodelling mechanism with most other chromatin remodellers. At the same time, differences between CHD4 and other chromatin remodellers result from the actions of auxiliary domains that regulate remodeller activity by for example: (1) making differential interactions with nucleosomal epitopes such as the acidic patch and the N-terminal tail of histone H4, and (2) inducing the formation of distinct multi-protein remodelling complexes (e.g. NuRD vs ChAHP). Thus, although we have learned much about remodeller activity, there is still clearly much more waiting to be revealed.

## Introduction

In its efforts to transcribe genes, RNA polymerase II (RNAPII) must deal with the histone octamers that package eukaryotic DNA as nucleosomes [1–5]. Although RNAPII can transcribe through a nucleosome [6], the re-establishment of chromatin structure following transcription requires a concerted effort between RNAPII itself, histone modifying enzymes, histone chaperones, and chromatin remodelling enzymes [4,6–11].

Chromatin remodelling enzymes use the energy derived from ATP hydrolysis to evict, assemble, alter the composition of, or reposition nucleosomes along chromatin [12]. There are four families of remodellers: chromodomain helicase DNA-binding (CHD), switch/sucrose non-fermentable (SWI/SNF), imitation switch (ISWI), and inositol requiring 80 (INO80) [12,13]. These enzymes (15 in humans, Table 1) all share a conserved Super Family 2 (SF2) ATPase domain that is the core DNA translocating motor [12]. Beyond this domain, they are differentiated by (sub)family-specific auxiliary domains and by their recruitment of other proteins to form multi-protein remodelling complexes [12].

**Table 1.** The human chromatin remodellers and their associated complexes

Family	Remodeller	Associated complexes
SWI/SNF	SMARCA2	cBAF, PBAF, ncBAF
	SMARCA4	
ISWI	SMARCA1	NURF, CERF, BRF, RSF, NORC, WICH, CHRAC, ACF
	SMARCA5	
CHD (subfamily I)	CHD1	
	CHD2	
CHD (subfamily II)	CHD3	NuRD
	CHD4	NuRD, ChAHP
	CHD5	NuRD
CHD (subfamily III)	CHD6	
	CHD7	
	CHD8	
	CHD9	
INO80	SRCAP	SRCAP complex
	EP400	TIP60/EP400 complex

Although unravelling of the mechanistic basis for chromatin remodelling has been a significant challenge, recent technological advances — perhaps most prominently in cryogenic electron microscopy (cryo-EM) and single-molecule biophysics — have allowed us to begin to peer inside the box to discover what makes remodellers tick. This review will focus on the nucleosome sliding mechanism of the essential remodeller CHD4, highlighting its emerging commonalities with other remodellers such as CHD1 and ISWI.

## CHD4 is an essential chromatin remodelling enzyme

CHD4 is a ubiquitous and abundant chromatin remodelling enzyme [14] that is essential for normal development across a range of organs and tissues [15–18]. CHD4 homologues are found in most complex organisms [19–23] and CHD4 function in humans is linked to many diseases, including acute myeloid leukaemia [24], rhabdomyosarcoma [25], endometrial carcinoma [26], and neurodevelopmental disorders [27,28]. CHD4 has also been identified as an essential gene in >1000 human cancer cell lines [29]. An understanding of CHD4 mechanism might, therefore, inform the development of therapeutics against such diseases.

Although it can function alone [30,31], CHD4 is also a core member of both the nucleosome remodelling and deacetylase (NuRD) complex and the ChAHP (CHD4, ADNP, HP1) complex [30,32,33]. Though originally (and often since) described as a transcriptional repressor [34–37] the NuRD complex has more recently been proposed to ‘fine-tune’ gene expression, acting as both an activator and a repressor to elicit subtle transcriptional changes [38]. In contrast, the ChAHP complex appears to repress lineage-specific genes and SINE elements by creating inaccessible heterochromatin [33,39]. Although participation in these complexes very likely modulates CHD4 function, very little is currently known about the nature of any such modulation. The following sections will, therefore, focus on the mechanism underlying the nucleosome remodelling activity of monomeric CHD4.

**Figure 1.**
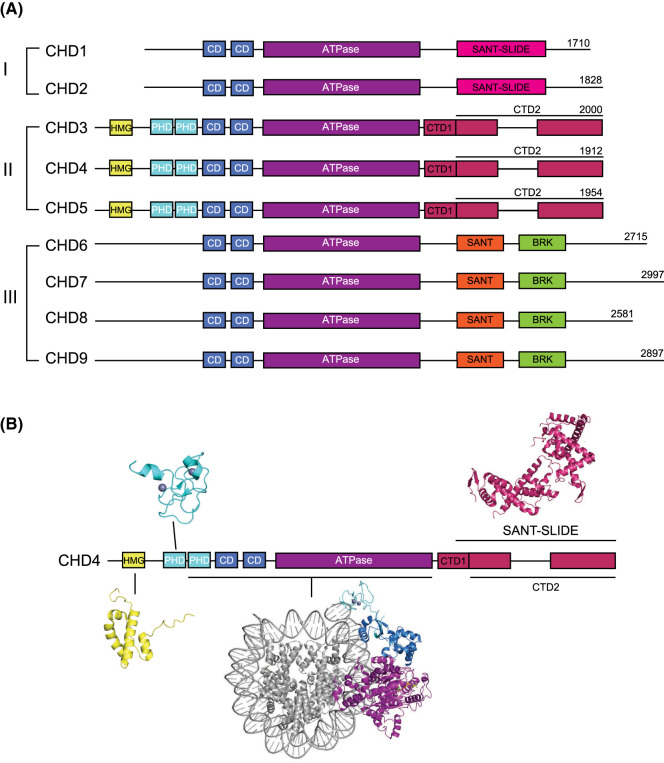
Domain architecture of human CHD-family remodellers. (**A**) Domain schematics of CHD1–9. Subfamily I comprises CHD1–2, subfamily II comprises CHD3–5, and subfamily III comprises CHD6–9. *HMG*; high mobility group, *PHD*: plant homeodomain, *CD*: chromodomain, *CTD*: C-terminal domain, *SANT*: Swi3/Ada2/N-Cor/TFIIIB domain, *SANT–SLIDE*: Swi3/Ada2/N-Cor/TFIIIB-SANT-like ISWI domain, *BRK*: BRM and KIS domain. Dashed lines indicate sequence not drawn to scale. (**B**) Available structural information on CHD4. The structures of the N-terminal high mobility group (HMG)-like domain (yellow, PDB: 2N5N), the PHD1 domain (cyan, PDB: 2L5U), the PHD2 domain (cyan, PDB: 2L75), CHD4 (residues 446–1200) bound to the nucleosome (purple, PDB:6RYR), and the C-terminal SANT–SLIDE domain (maroon, PDB: 8D4Y) are indicated together with a schematic of CHD4 domain architecture.

The CHD remodellers are grouped into three subfamilies based on their complement of auxiliary domains (Figure 1A). CHD4 belongs to CHD subfamily II, together with CHD3 and CHD5. In addition to the ATPase, CHD4 harbours a DNA-binding high mobility group (HMG) box-like domain, tandem histone-binding plant homeodomain (PHD) domains, tandem DNA-binding chromodomains, several autoinhibitory regions, and a newly identified DNA-binding SANT–SLIDE domain [40–44] (Figure 1B).

Because CHD4 does not have a yeast homologue, it has proven more challenging to work with than many other remodellers and so we begin by describing the evolution of our understanding about remodeller mechanism from work carried out mainly on remodellers found in *Saccharomyces cerevisiae*.

## What have we learned about chromatin remodellers?

The DNA translocating motor common to all chromatin remodellers comprises two RecA-like lobes. RecA is a bacterial DNA recombination repair protein that was found to have unexpected structural homology to the ATPase domain of SF2 helicases [45–48]. The ATP binding site of the RecA-like fold is formed by conserved motifs that formed the basis from which chromatin remodellers were initially classified as helicases. However, because remodellers lack the ability to separate DNA strands they are now known to be double-stranded DNA translocases [45–48]. Structural homology between RecA and the ATPase domain of SF2 helicases suggested a common ATP binding and hydrolysis mechanism that drives changes to the relative orientation of the two RecA-like lobes [45,49]. These conformational changes ultimately move the helicase relative to the nucleic acid substrate [45,49]. In the context of the nucleosome with the remodeller in a fixed position, the DNA would instead be translocated around the nucleosome [12]. However, because no remodeller-nucleosome structures existed at the time, the mechanism by which DNA might be propagated around the nucleosome was unclear.

**Figure 2.**
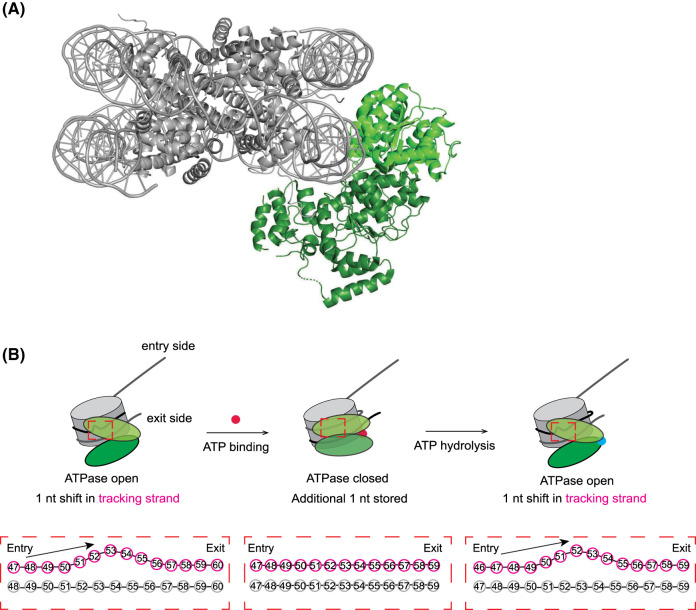
Structure of Snf2 bound to the nucleosome and simple model of the remodelling mechanism based on structural snapshots. (**A**) Cryo-EM structure of Snf2 (green) bound to the nucleosome (grey) (PDB: 5X0Y). (**B**) Chromatin remodelling mechanism derived from cryo-EM structures. The ATPase binds to the nucleosome in an open conformation in the absence of ATP and induces a 1-nt DNA distortion of the DNA tracking strand (pink, inset below). Binding of ATP (red) (red circle) results in a closed conformation of the ATPase and movement of the other DNA strand (guide strand, grey), with the overall effect of moving the DNA 1 bp in the indicated direction. ATP hydrolysis (to ADP, blue circle) resets the ATPase to an open conformation and again induces a 1-nt DNA distortion of the tracking strand. The translocated base pair is somehow temporarily absorbed into the nucleosome.

A mechanism began to emerge in 2017 with the cryo-EM structure of the yeast Snf2 remodeller bound to a nucleosome (Figure 2A) [50]. In this structure, the two lobes of the Snf2 ATPase (in the absence of a bound nucleotide) bind the nucleosome at superhelical location +2 (SHL + 2) in what has been called the ‘open' conformation. Strikingly, Snf2 binding induces, at the remodeller binding site, a 1-nucleotide (nt) shift of one strand of the DNA (the so-called tracking strand) towards the dyad. This shift, the energetic cost of which is paid simply by Snf2 binding, is propagated upstream all the way to the DNA entry side, although canonical base pairing is maintained. DNA on the dyad side of SHL + 2, however, is unperturbed. The net result is that the tracking strand is drawn into the nucleosome by a distortion of the DNA, suggesting this is the first step in a mechanism that translocates DNA relative to the histone octamer.

Two subsequent structures of Snf2–nucleosome complexes [51] filled out the story substantially. A structure of Snf2 bound to ADP closely resembled the apo-structure, with the tracking strand again advanced towards the dyad by 1 nt. In contrast, the binding of an ATP mimic was seen to rotate lobe 2 of the ATPase into a ‘closed' conformation that leaves the nucleosomal DNA in its canonical position. A flurry of additional remodeller-nucleosome structures in various nucleotide-bound states ensued, revealing that most remodellers engage the nucleosome at SHL + 2, induce the same 1-nt distortion of the tracking strand at the binding site (when in either the apo- or the ADP-bound form), and undergo the same ATPase lobe rearrangements in different nucleotide-bound states [52–58]. An exception to this trend is INO80, which binds to the nucleosome at SHL-6 and has not been shown to induce the same nucleosomal DNA distortions [59,60]. This may relate to its primary role in histone dimer exchange in contrast to the nucleosome sliding function of CHD, ISWI, and SWI/SNF family remodellers [59,60].

These structures have led to the proposal of a general mechanism for nucleosome sliding in which binding of the apo-enzyme to a nucleosome shifts the tracking strand by 1 nt and binding of ATP then advances the guide strand similarly (Figure 2B). This series of events effectively stores an additional base pair in the nucleosome. Although the manner in which it is stored is not currently known, nucleosome structures have been determined that package 145, 146, or 147 bp of DNA [61,62], suggesting some ability of the histone octamer to accommodate a range of sequence lengths. ATP hydrolysis (generating the ADP-bound state and subsequently the apo state) shifts the tracking strand a second time and the cycle can then continue, allowing processive movement of the DNA relative to the histone octamer.

## CHD4 employs a conserved nucleosome sliding mechanism

These insights into remodeller mechanism developed over nearly 20 years, with the structural work supported by biophysical and biochemical studies on multiple enzymes [63–66]. In contrast, the activity of CHD4 remained largely unstudied during this time. More recently, a combination of the cryo-EM structure of a CHD4-nucleosome complex [44] with single-molecule and other fluorescence remodelling assays [31,41] has shown that CHD4 follows firmly in the mechanistic footsteps of other chromatin remodellers.

**Figure 3.**
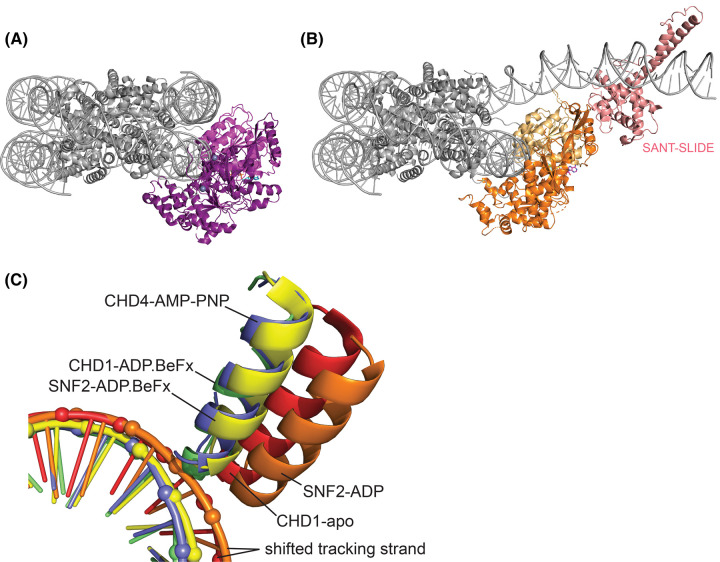
The mode of CHD4-DNA interaction is consistent with that observed for other remodeller complexes. (**A**) Cryo-EM structure of CHD4 bound to the nucleosome (PDB: 6RYR). (**B**) Cryo-EM structure of CHD1 bound to the nucleosome (PDB: 5O9G). Binding of the CTD to DNA induces a significant opening of the nucleosome. (**C**) Overlay of the structures of a nucleosome bound to: SNF2 in the presence of ADP (dark green, 5Z3O), apo-CHD1 (orange, 7TN2), SNF2 in the presence of ADP.BeFx (light green, 5Z3U), CHD1 in the presence of ADP.BeFx (yellow, 5O9G) and CHD4 in the presence of AMP-PNP (purple, 6RYR). Structures are overlaid over the histone octamer. Positioning of the gating helix of ATPase lobe 2 reflects the nucleotide-bound state, even between different remodellers from different species. CHD4, SNF2, and CHD1 are observed in the closed conformation when bound to ATP mimics and the DNA path resembles that of an unbound nucleosome. Movement of the tracking strand is observed only for the apo or ADP-bound complexes in which SNF2 or CHD1 is in the open conformation (red, orange).

The CHD4-nucleosome structure (Figure 3A) demonstrates that CHD4, like other remodellers, binds to the nucleosome at SHL + 2 [44,53,54]. Despite the similarity in the binding mode of the ATPase between CHD4 and its paralogue CHD1 (Figure 3B), the behaviour of the C-terminal region is strikingly different. Multiple structures of CHD1 [52,54,67] show that the C-terminal SANT–SLIDE domain binds exit-side DNA, peeling **∼**20 nt away from the nucleosome surface. In contrast, although CHD4 also carries a corresponding SANT–SLIDE domain [41], this region of the protein was not observed in the structure and the DNA was not perturbed in the manner seen for the CHD1 complex. The reason for this difference is not clear.

The CHD4-nucleosome structure was determined in the presence of the ATP mimic AMP-PNP and shows the ATPase in the closed conformation, closely matching the conformation observed for the corresponding Snf2 and CHD1 complexes (Figure 3A,B). Comparison of these structures with the apo-CHD1 and ADP-bound Snf2 structures suggests that only the open conformation taken up in the apo-ADP-bound states is sterically compatible with the tracking strand shift, as previously noted [52]. Although no ADP-bound structure of CHD4 is currently available, this comparison is consistent with the idea that the CHD4 ATPase translocates DNA using the same core mechanism as other remodellers. It seems likely that this mechanism will prove to be widely conserved.

In addition to providing a snapshot into the mechanism of chromatin remodelling by CHD4, the CHD4-nucleosome structure also demonstrates how disease-associated mutations can impact CHD4 remodelling activity and function [44]. Mutations in ATPase lobe 1 (S851Y) and ATPase lobe 2 (G1003D, R1068H, R1127N, and W1148L) have been linked to a neurodevelopmental disorder [27,68]. The CHD4-nucleosome structure shows that these mutations in the ATPase lobes would likely impact the stability of the ATPase fold and disrupt interactions with the nucleosome [44]. Indeed, the W1148L mutation in ATPase lobe 2 has been shown to decrease remodelling activity [28]. Similarly, numerous missense mutations in the ATPase domain that reduce remodelling activity are linked to endometrial cancer [69]. Interestingly, a mutation — H1196Y — at the boundary of the ATPase domain and the CTD1 region increases CHD4 remodelling activity *in vitro* [69], in line with data showing that this region is autoinhibitory [41].

Although the structures described above have provided enormous insight, their static nature leaves many mechanistic questions unanswered; such questions can be addressed by biochemical assays that observe remodelling in real time. Single-molecule fluorescence resonance energy transfer (smFRET) assays allow the nucleosome sliding activity of individual chromatin remodeller molecules to be measured as a function of time [70–73]. Recent application of this approach to CHD4 [31] demonstrated that the movement of DNA into the nucleosome (at the entry side) is decoupled from the ejection of DNA from the exit side. CHD4 was shown to draw DNA into the nucleosome continuously — most likely in 1-bp steps — but expel DNA from the exit side in discrete 4–6 bp segments. Similar observations have been made for CHD1, SNF2h, and RSC [72,74,75]. Quantitative analysis of CHD4 smFRET data [31] further indicated that (1) 4–6 identical reaction steps take place prior to the expulsion of DNA and (2) the rate of these steps is dependent on ATP concentration. It was also notable that the time between successive DNA expulsion events was independent of CHD4 concentration, supporting the idea that remodeller activity is processive. Finally, it was observed that even the binding of CHD4 to the nucleosome in the absence of ATP gave rise to movement of entry-side DNA, in line with the idea that the binding energy from remodeller binding alone is sufficient to stimulate some translocation of DNA into the nucleosome.

Taken together, these data point to a model for nucleosome sliding in which the cycle depicted in Figure 2B is repeated until 4–6 bp of DNA are absorbed into the nucleosome and ‘stored’ somewhere (in an unknown manner) between the CHD4 binding site at SHL + 2 and the exit side. At this point, the strain of such a conformation is resolved by the expulsion of most or all of this extra DNA.

## Regulation of CHD4 activity by auxiliary domains

Despite the broad conservation of a core remodelling mechanism across remodeller families and across evolution, the functional outcome of this DNA translocation appears to depend on the identity of the remodeller. For example, in *in vitro* nucleosome sliding assays, CHD1, ISWI, and INO80 remodellers position nucleosomes towards the centre of a length of DNA and thus generate regularly spaced nucleosome arrays [76–80], whereas RSC and CHD4 exhibit some preference for positioning histone octamers towards the ends of DNA [31,66]. How is the activity of each remodeller regulated to produce distinct remodelling outcomes? For CHD1 and ISWI remodellers, this question is partly addressed by the presence of autoregulatory domains, suggesting that this might also be the case for CHD4 [12,81,82]. Evolutionary conservation of these auxiliary domains in CHD4 underscores their functional importance — the chromodomains and C-terminal domains are conserved from sponge to human, and the HMG, PHD, chromodomains, and C-terminal domains are conserved from fly to human [19–23].

In isolation, the ATPase domain of CHD4 has impaired remodelling activity compared with the full-length protein, indicating that the surrounding domains contribute to remodelling activity [43]. Remodelling assays carried out with CHD4 truncations further support this idea [41]. Unfortunately, more than two-thirds of CHD4 was not observed in the CHD4-nucleosome structure (residues 1–445 and ∼1200–1912), meaning that functional inferences need to come from biochemical approaches.

At the N-terminal end, a truncation lacking the N-terminal intrinsically disordered region [IDR1, CHD4(1–145)] displays slightly higher remodelling activity than WT CHD4 [41], suggesting this region is mildly autoinhibitory, although the mechanism underlying this effect is not known. In contrast, further truncation — of the HMG-like domain and the following IDR (IDR2) — decreases the rate of remodelling three-fold. The HMG-like domain can bind DNA with moderate affinity [40], though not in the sequence-specific manner displayed by the bona fide DNA-binding HMG domains found in HMGB and SOX proteins. It is notable that HMG domains induce DNA bending, even in the context of the nucleosome [83–85]. Structures of the SOX2-nucleosome complex show the SOX2 lifting DNA away from the histone octamer [84,85], raising the possibility that the CHD4 HMG-like domain might stabilise intermediates in the chromatin remodelling cycle that have ‘stored’ several base pairs of DNA. Indeed, it has been speculated that HMGB proteins might enhance chromatin remodelling by making interactions of this type [86].

IDR2 (residues 215–354) links the HMG-like domain with tandem PHD domains. Although scrambling the sequence of this region had no impact on remodelling, halving the number of basic residues (from 32 to 15) halved the rate of remodelling [41]. IDR2 contains motifs that are found in H1 linker histones (proline/lysine-rich motifs), where they stabilise binding of H1 to the dyad and entry/exit sites of the nucleosome. It IDR2 also harbours a di-arginine-containing motif that resembles the histone H4 N-terminal tail. A similar sequence in several other remodellers (including CHD7 [87]) promotes activity by binding the nucleosome acidic patch [88,89], thereby and displacing the H4 N-terminal tail, which in turn binds an acidic surface in the ATPase [50,54,90]. This latter interaction is also seen in the CHD4-nucleosome structure [44]. Overall, these data suggest that IDR2 contributes to remodelling activity by contacting the nucleosome both at the acidic patch and at the entry/exit sites. Such interactions are likely to be conserved among diverse remodellers.

The C-terminal region of remodellers such as CHD1 and ISWI features a tripartite DNA-binding HAND–SANT–SLIDE (HSS) domain. These proteins also harbour a ∼60-residue autoinhibitory motif (the bridge in CHD1 [81] and NegC in ISWI [91]) that binds lobe 2 of the ATPase domain in a meandering fashion, holding it in a very open conformation that cannot fully engage the DNA at SHL + 2. It has been proposed that the HSS domain acts as a substrate sensor that recognises extranucleosomal (linker) DNA, inducing a structural rearrangement that releases the autoinhibitory motif to promote remodelling activity [52,81,90–92].

As noted above, CHD4 also harbours a C-terminal SANT–SLIDE domain (residues 1380–1810) that can bind DNA [41]. Deletion of this domain reduces the affinity of CHD4 for a nucleosome by 10-fold and the rate of remodelling six-fold, demonstrating its functional importance. In contrast, further deletion of residues 1231–1379 [CTD1 in Figure 1B, creating CHD4(1–1230)] *raises* the remodelling rate two-fold over WT CHD4, identifying CTD1 as a strong autoinhibitory motif. Mutations in the corresponding region of the Drosophila CHD4 homologue similarly increase activity [69]. This region has distant homology to the CHD1 bridge and cross-linking-mass spectrometry data confirm that it contacts the same surface of the ATPase as the bridge/NegC motifs [41]. Thus, CHD1, CHD4 and ISWI proteins (as well as CHD3 and -5) likely share this C-terminal regulatory unit comprising an autoinhibitory motif and a substrate sensor that releases autoinhibition, demonstrating further mechanistic commonality amongst these proteins.

## Regulation of CHD4 activity by the nucleosome substrate

How do features of the nucleosome itself contribute to CHD4 activity? There are several structural cues that have been demonstrated to be important in the activity of chromatin remodelling enzymes, including the acidic patch formed by H2A and H2B and a basic motif present on the N-terminal H4 tail.

**Figure 4.**
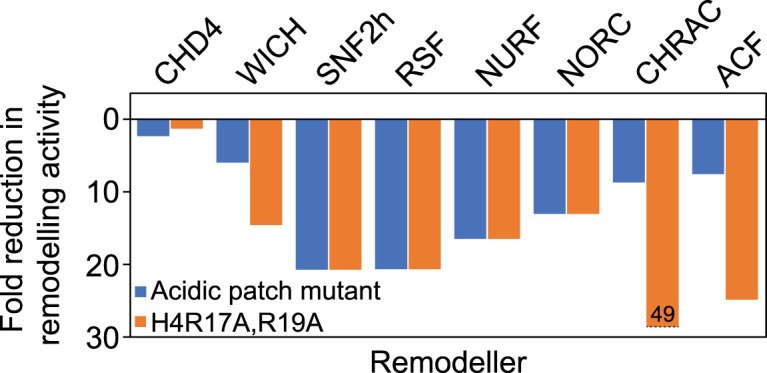
CHD4 activity is less reliant than other remodellers on the nucleosome acidic patch and the H4 arginine anchor motif. Fold reduction in the rate of nucleosome sliding activity for seven remodelling enzymes/complexes following either: (1) mutation of the nucleosome acidic patch (blue bars) or (2) mutation of the arginine anchor motif in the N-terminal tail of histone H4 (orange bars).

The acidic patch is a cluster of glutamates and aspartates on H2A/H2B that has emerged as an important binding site on the nucleosome surface for many chromatin regulatory proteins [93–95]. Many remodellers are highly sensitive to acidic patch mutations [94,96], a trend that was emphasised in pioneering work from the Muir lab that dissected the effect of >100 variant nucleosomes on the activity of eight diverse remodellers [94]. Strikingly, acidic patch mutations reduced activity by 6- to 19-fold for all remodellers other than CHD4 (Figure 4). CHD4 displayed only a two-fold reduction, suggesting that this is a less prominent means of regulating CHD4 function. Similarly, it appears that the acidic patch is not essential for CHD1 remodelling activity [97].

As discussed earlier, the N-terminal H4 tail plays a prominent role in regulating chromatin remodeller activity. The H4 tail contains a basic motif (residues R17, H18, R19, and K20) that inserts into an acidic cavity in the ATPase of many remodellers, including ISWI, CHD1, and CHD4 [44,53,54,91]. In ISWI, for example, the autoinhibitory AutoN region binds this cavity in the apo remodeller, maintaining a conformation with low ATP hydrolysis activity [90,91]. Upon binding to the nucleosome, the H4 tail displaces AutoN, which in turn binds the acidic patch; together, these rearrangements stimulate ISWI remodelling activity [90,98]. In line with these observations, mutation of H4 R17/R19 severely impairs the remodelling activity of ISWI [91,94] and many other [94] remodellers (Figure 4). Perhaps surprisingly, given the structural similarities noted above, the effect of these H4 mutations on CHD4 remodelling activity is small (∼1.5-fold, Figure 4, [94]), suggesting that this is a less important aspect of CHD4 regulation. In line with observations made for CHD4, INO80 remodelling activity is not affected by the presence of the H4 tail [60].

The activity of CHD4 (at least in *in vitro* nucleosome sliding assays) thus appears significantly less dependent than many other remodellers on two nucleosome structural features: the histone H4 tail and the acidic patch. Although the molecular basis for this difference is unknown, it might hint at a distinct biological function for CHD4.

Another important substrate-related question is that of how CHD4 (and other chromatin remodelling enzymes) chooses its targets among the tens of millions of nucleosomes packaging a complex genome? Histone post-translational modifications (PTMs) and variants clearly have the capacity to impact both the affinity of a remodeller for a substrate and its activity on the substrate [95].

A recent tour de force of chromatin biochemistry assessed the relative affinity of ∼2000 nuclear proteins for >80 distinctly modified dinucleosomes [99]. With the caveat that observations for CHD4 will be modulated by its association with other members of the NuRD complex (e.g. MBD2 selectively binds methylated DNA), these data indicate that CHD4 binds preferentially to nucleosomes containing: (1) jointly acetylated H3K9 (H3K9ac) and H3K14, or (2) monomethylated H3K4 (H3K4me1), with a reciprocal dislike of trimethylated H3K4 [99]. The effects of H3K9ac and H3K4me3 are in line with known binding preferences of the CHD4 PHD domains [42,100,101]. In contrast, the preference for binding H3K4me1 — a mark associated with enhancers — had not been previously reported, although recent ChIP-seq data associated CHD4 more strongly with enhancers than promoters [25]. It is worth noting that the binding preferences reported in this study are not fully recapitulated in the activity data reported by the Muir group [94]. In the latter work, neither H3K9ac nor H3K4me1 increase CHD4 activity. While these differences could arise from assay differences (e.g. the use of recombinant versus native CHD4), they might also reflect a distinction between factors that control the targeting of CHD4 versus factors that regulate catalytic activity.

**Figure 5.**
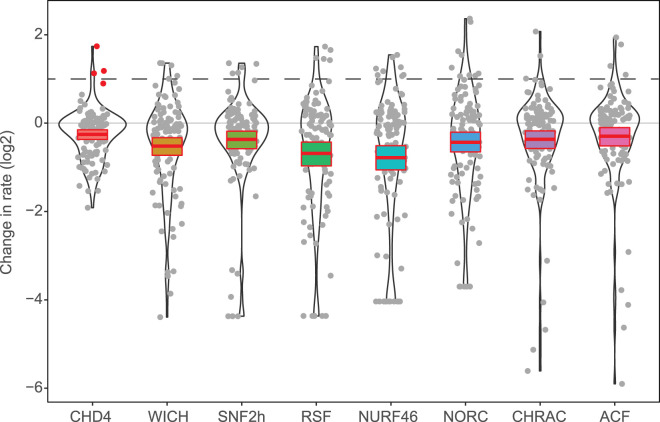
Effect of histone PTMs and sequence changes on *in vitro* nucleosome remodelling rate. Data are taken from Dann et al. [94] and are plotted as log(2) fold changes. The nucleosome sliding activity was measured for an unmodified nucleosome, together with 111 variants that include common histone PTMs, histone variants and several previously identified histone mutants. The mean and standard deviation are indicated for each of the eight remodellers tested and the dotted line indicates a two-fold increase in activity compared with unmodified nucleosome. Red data points indicate histone mutants.

Another striking feature of the Muir dataset is that, although numerous PTMs decrease the CHD4 remodelling rate, none of the surveyed PTMs or naturally occurring histone variants increased CHD4 activity by more than ∼1.5-fold (Figure 5). This observation suggests that common elements of the histone code do not exert their influence by directly increasing the CHD4 catalytic rate. Indeed, rather few PTMs increased the catalytic rate of any of the eight remodellers tested.

Extranucleosomal DNA can also influence both remodelling activity and directionality. CHD1, ISWI, and INO80 remodellers can sense extranucleosomal DNA length such that their remodelling activity increases with increasing DNA length up to ∼60 bp [80,102,103]. It is proposed that these remodellers use extranucleosomal DNA to act as a ‘protein ruler’ to set the spacing between adjacent nucleosomes [79,102,103]. In the case of CHD1, the DNA-binding SANT–SLIDE domain interacts with extranucleosomal DNA and is required for nucleosome centring [63,78,92]. Impairment of this interaction results in bi-directional nucleosome sliding, suggesting a role for the SANT–SLIDE domain in remodelling fidelity [104]. In contrast with CHD1, ISWI, and INO80 remodellers, CHD4 is more active on nucleosomes with symmetrical extranucleosomal DNA [31]. Although the basis for this difference is not known, it might again hint at differences in CHD4 function — perhaps creating nucleosome-depleted and nucleosome-rich regions rather than regularly spaced arrays. Consistent with this idea, chromatin accessibility assays demonstrate that CHD4 can both promote DNA accessibility at enhancers [25] and establish inaccessible chromatin [105–107].

## Concluding remarks

CHD4 slides nucleosomal DNA along the histone octamer in short ∼5-bp bursts [31]. A similar sliding mechanism is observed in CHD, ISWI, and SWI/SNF remodellers, suggesting core similarities in remodelling mechanism across chromatin remodeller families [70,71]. There also appear to be differences in the way that these remodellers operate. For example, in CHD1, ISWI, and INO80 remodellers slide nucleosomes towards the centre of DNA to create evenly spaced nucleosome arrays [78,79], whereas CHD4 and RSC may prefer, at least *in vitro*, to slide nucleosomes towards the ends of DNA [25,31,105].

An important aspect of CHD4 function is the mechanism by which the enzyme is recruited to target genes. CHD4, CHD1, and ISWI remodellers all feature auxiliary domains that complement the basic sliding activity of the translocase domain and guide remodelling activity to the appropriate nucleosome substrate [41,81,91]. Autoinhibitory domains also regulate the ATPase activity of the translocase, and although the details are not yet clear the mechanism controlling release of autoinhibition appears to be at least partially distinct for CHD4 and ISWI remodellers. For example, autoinhibition by the ISWI AutoN domain is relieved through interaction with the H4 tail basic patch and the H2A–H2B acidic patch [91,94,96], whereas these nucleosomal epitopes are only mildly stimulatory towards CHD4 remodelling activity [94]. Similarly, the HMG-like domain of CHD4 is found in CHD3 and CHD5, but no other remodellers, hinting at functional differences even within the CHD family.

In addition to the role of nucleosomal features such as histone PTMs (discussed above), CHD4 is likely recruited to target genes by transcription factors and other chromatin ‘reader’ proteins. For example, CHD4 has already been shown to interact with transcription factors such as ADNP [33], GATA4/NKX2–5/TBX5 [108], ZFHX4 [109], and HIF1/2 [110]. Additionally, CHD4 is known to interact with the histone acetylation reader proteins BRD3 and BRD4 [111,112]. Based on these latter interactions, it might be expected that BRD3 and BRD4 recruit CHD4 to acetylated regions of the genome, which is notable in light of the fact that the NuRD complex features two histone deacetylases as core subunits. An interesting question is that of whether interactions with transcription factors and reader proteins also directly affect the remodelling activity of CHD4 (rather than just its recruitment), either through perturbation of nucleosome structure or via conformational changes to and release of autoinhibitory elements of CHD4.

Overall, significant advances have been made in the last 5 years in our mechanistic understanding of CHD4 activity — due to work both on CHD4 and on other chromatin remodelling enzymes. Despite these advances, there is a lot more to learn. The precise roles of N- and C-terminal domains are yet to be elucidated, and at a fundamental level, we really lack a clear view of the biochemical consequences of CHD4 action *in vivo*. There is plenty more to do.

## Perspectives

- The packaging of DNA by nucleosomes is inhibitory to processes such as transcription, DNA repair, and replication. CHD4 is an essential chromatin remodelling enzyme that regulates access of cellular machinery to genomic DNA. Recent work has provided significant insight into the mechanisms underlying CHD4 activity.
- Work on both CHD4 and other chromatin remodelling enzymes suggests that there are both strong commonalities and clear differences in the mechanism underlying the activity of CHD4 and other remodellers.
- Key unanswered questions include how auxiliary domains regulate CHD4 activity, how CHD4 is targeted to specific genomic target sites, and how the movement of histone octamers relative to DNA actually alters gene transcription.

## Abbreviations

CHD: chromodomain helicase DNA-binding
cryo-EM: cryogenic electron microscopy
HMG: high mobility group
HSS: HAND–SANT–SLIDE
INO80: inositol requiring 80
NuRD: nucleosome remodelling and deacetylase
RNAPII: RNA polymerase II
SF2: Super Family 2
SHL + 2: superhelical location +2
smFRET: single-molecule fluorescence resonance energy transfer
SWI/SNF: switch/sucrose non-fermentable
